# Contemporary perspectives on metabolic surgery for type 2 diabetes: surgical techniques, outcomes, and complications

**DOI:** 10.3389/fendo.2026.1849157

**Published:** 2026-07-01

**Authors:** Jian Chen, Wei Liu, Yuxi Bai, Cheng Jiao

**Affiliations:** 1 Department of General Surgery, Bethune International Peace Hospital, Shijiazhuang, Hebei, China; 2 Department of Neurology, Second Hospital of Hebei Medical University, Shijiazhuang, Hebei, China

**Keywords:** diabetes, diabetes remission, efficacy, metabolic mechanisms, metabolic surgery, postoperative complications, surgical procedures

## Abstract

Metabolic surgery has emerged as an important therapeutic option for the management of type 2 diabetes mellitus (T2DM) and obesity and has gained increasing clinical attention in recent years. Ongoing research has expanded the understanding of various surgical techniques and their therapeutic efficacy. Nevertheless, concerns related to postoperative diabetes recurrence and the prevention and management of procedure-related complications continue to constrain the routine application of metabolic surgery in T2DM care. This review provides a systematic overview of the principal metabolic surgical procedures primarily used in the management of T2DM, compares their efficacy, and examines the incidence and management of associated complications. In addition, preoperative predictors of surgical outcomes are evaluated, and long-term postoperative effectiveness is reviewed. By synthesizing recent evidence, this review aims to offer clinicians and researchers a coherent theoretical framework and practical insights to support the incorporation of metabolic surgery into comprehensive multimodal strategies for diabetes management.

## 1 Introduction

Type 2 diabetes mellitus (T2DM) is among the most prevalent chronic conditions globally, with incidence rates continuing to rise. Research has indicated a strong association between its development and factors such as unhealthy lifestyle behaviors, dietary patterns, and obesity, as well as notable comorbidities, including cardiovascular diseases, malignancies, and autoimmune disorders (1, 2). Given its increasing prevalence across populations, T2DM is regarded as a significant public health concern, underscoring the urgent need for effective prevention and treatment strategies.

Conventional management strategies for diabetes have traditionally included glucose-lowering pharmacotherapies and insulin replacement therapy. In recent years, specific surgical interventions have demonstrated effectiveness in lowering blood glucose levels and controlling body weight, with substantial clinical evidence supporting the therapeutic efficacy of metabolic surgery in the management of diabetes. Metabolic surgery induces significant weight loss in patients, ameliorates obesity-related metabolic abnormalities, and facilitates diabetes remission through multiple physiological mechanisms, thereby improving clinical outcomes (3). These benefits are particularly relevant for patients who demonstrate inadequate responses to standard pharmacological treatments, for whom metabolic surgery represents an alternative therapeutic option (4).

Pharmacological therapy is frequently limited in patients with medication resistance, and prolonged medication use may be associated with adverse effects that negatively affect treatment adherence (5). Metabolic surgery primarily exerts its effects through reconstruction of the gastrointestinal tract, which triggers a cascade of multifaceted physiological alterations. These include changes in nutrient absorption patterns and efficiency, as well as modulations of gut hormone secretion, gut-brain axis signaling, bile acid metabolism, and gut microbiota composition-all of which are active areas of ongoing research and not yet fully elucidated. This approach circumvents adverse effects and drug resistance associated with pharmacological therapy, fundamentally improves glucose metabolism, reduces the risk of diabetes-related complications, and contributes to an improved long-term prognosis (6, 7).

In recent years, debate within the academic community has focused on the clinical application of metabolic surgery, particularly regarding the selection of surgical procedures and the long-term outcomes associated with these interventions (4, 8). Therefore, a thorough understanding of the current landscape of metabolic surgery is essential. This includes the synthesis of existing research findings and the provision of a comprehensive and objective evaluation of the various procedures with respect to surgical techniques, therapeutic efficacy, and associated complications, thereby informing the direction of future clinical research.

## 2 Main surgical procedures of metabolic surgery

Metabolic surgical procedures used in clinical practice primarily consist of Roux-en-Y gastric bypass (RYGB), sleeve gastrectomy (SG), and biliopancreatic diversion with duodenal switch (BPD/DS), along with newer or modified variants of these techniques.

### 2.1 Roux-en-Y gastric bypass

RYGB is a metabolic surgical procedure widely implemented for the management of obesity and T2DM. The procedure involves the creation of a small gastric pouch that is anastomosed to the proximal jejunum, thereby bypassing most of the stomach, the entire duodenum, and the proximal jejunum. Through a reduction in gastric volume and alteration of intestinal anatomy, a significant decrease in food intake is achieved. In addition, rerouting of the digestive pathway is associated with enhanced metabolic effects, improved insulin sensitivity, and better glycemic control, likely through multiple complementary mechanisms-including modulation of the gut-brain vagal axis and its influence on central brain centers that regulate energy homeostasis.

RYGB is indicated for patients with severe obesity (body mass index [BMI] ≥ 40 kg/m²) or for those with a BMI between 30 and 40 kg/m² with comorbid conditions including type 2 diabetes or hypertension (9).

Reductions in fasting plasma glucose, glycated hemoglobin (HbA1c), and blood lipid levels have been observed following RYGB. The procedure is associated with improvements in insulin resistance, along with multiple complementary metabolic effects-including restoration of first-phase insulin secretion, reduced glucagon secretion, attenuated hepatic gluconeogenesis, and enhanced glucose sensitivity of brain centers. Collectively, these changes have resulted in complete remission of T2DM in 65.2% of patients within six months postoperatively (9). Data from a large-scale cohort study conducted by the ARMMS-T2D consortium show that the complete remission rate of type 2 diabetes can reach 65%–74% within 6 months after RYGB surgery (10). Longer-term follow-up data indicate that, one year after RYGB, 60%–80% excess weight loss is typically achieved, with mean BMI decreasing from 40 kg/m² to approximately 28–30 kg/m², representing a reduction of 10%–12% (11).

The metabolic effects of RYGB are thought to be linked to changes in gut hormone secretion and gut microbiota composition (12, 13), with glucagon-like peptide-1 (GLP-1) and peptide YY being among the most widely studied gut hormones. These hormones are regulated by neural signaling pathways activated upon nutrient stimulation of specific intestinal segments. Following metabolic surgery, modifications to gastrointestinal anatomy alter the pattern of nutrient stimulation in hormone-secreting regions, thereby enhancing insulin secretion, suppressing appetite, and improving overall metabolic function. Notably, emerging evidence indicates that the metabolic benefits of bariatric surgery can occur independently of GLP 1 signaling, as demonstrated in studies showing efficacy in GLP 1 receptor deficient animal models and suggesting that gut hormone changes may not be an absolute requirement for therapeutic success (14–16). Furthermore, RYGB associated favorable alterations in gut microbiota have been closely associated with improved glycemic control in patients with diabetes (13, 14).

Common postoperative complications associated with RYGB include anastomotic leak, hemorrhage, deep vein thrombosis, nutritional deficiencies, internal hernia, and anastomotic ulceration (17). Long-term follow-up studies have indicated that sustained glycemic control following RYGB is influenced not only by the surgical intervention itself but also by multiple contributing factors, including lifestyle modifications and dietary management. Therefore, thorough preoperative assessment of the clinical condition of each patient is essential to support informed and appropriate surgical decision-making (18, 19).

### 2.2 Sleeve gastrectomy

SG is a relatively recent metabolic surgical procedure that has been increasingly adopted worldwide in recent years. The procedure involves resection of the greater curvature and fundus of the stomach, removing approximately 70%–80% of the gastric tissue and resulting in the formation of a narrow, tube-shaped sleeve along the lesser curvature. This modification reduces gastric volume to approximately 50–150 mL, thereby limiting food intake. In addition, changes in the secretion of hormones including ghrelin contribute to appetite suppression and promote weight loss. SG has received growing clinical attention due to its technical simplicity, shorter recovery time, and comparatively lower complication rate (20). SG is primarily indicated for patients with obesity and coexisting T2DM, particularly those for whom conservative management has failed to achieve significant weight reduction. Postoperative reductions in visceral fat following SG have been associated with substantial weight loss and improvements in glycemic control (21).

Currently, most studies indicate that RYGB is associated with superior long-term outcomes in weight loss and diabetes remission when compared with SG (22–24). At one year postoperatively, remission of T2DM has been observed in approximately 50% of patients undergoing RYGB, whereas the corresponding rate for SG is approximately 20% (22–24).

The primary advantages of SG include lower surgical risk, a reduced incidence of complications, faster postoperative recovery, and a lower likelihood of developing nutritional deficiencies (23). As a technically straightforward and safe procedure, particularly when performed laparoscopically, SG offers reduced operative complexity when compared with other bariatric procedures. Consequently, it has become a preferred option for bariatric surgery and is increasingly adopted in clinical practice, particularly among patients seeking to avoid more complex surgical interventions (22).

### 2.3 Biliopancreatic diversion with duodenal switch and other emerging procedures

BPD/DS is recognized as an effective metabolic surgical procedure that incorporates technical elements of both SG and RYGB. The procedure involves performing a distal gastrectomy to restrict food intake, along with transection of the small intestine approximately 300 cm proximal to the ileocecal valve. The proximal portion of the small bowel is anastomosed to the ileum approximately 50 cm from the ileocecal valve, and the remaining stomach is connected to the distal small intestine. By limiting gastric capacity and rerouting the passage of ingested nutrients through the gastrointestinal tract, BPD/DS facilitates substantial weight loss and significant metabolic improvements.

Research has reported that patients undergoing BPD/DS achieve an average excess weight loss of 62% ± 23.03% within five years postoperatively, with remission rates of diabetes, hypertension, and dyslipidemia reported at 96%, 77%, and 84%, respectively (25). Compared with RYGB, BPD/DS has been shown to result in a significantly higher excess weight loss of 85%–95% at one year postoperatively, versus 65%–75% with RYGB, along with an average BMI reduction of 18%–20%. At the 10-year follow-up, 75%–85% of patients maintained an excess weight loss of more than 70%, and 95% of patients achieved diabetes remission and no longer required glucose-lowering medications (26). Compared with RYGB, BPD/DS has demonstrated superior long-term weight loss and glycemic control in randomized controlled trials with 10-year follow-up (27).

The primary adverse effects associated with BPD/DS include steatorrhea, chronic diarrhea, hepatic dysfunction, anastomotic leak, and internal hernia. In some cases, severe complications including gastrointestinal fistulae may occur and can be life-threatening (28). Therefore, despite its notable long-term efficacy in promoting weight loss and metabolic improvement, BPD/DS continues to be approached with caution by many surgeons due to its technical complexity and the considerable risk of postoperative nutritional complications (25, 29).

In recent years, single-anastomosis duodeno-ileal bypass with sleeve gastrectomy (SADI-S), a simplified variant of BPD/DS, has gained significant clinical attention. This procedure modifies the traditional BPD/DS by incorporating a single anastomosis. Favorable outcomes have been reported with SADI-S in terms of weight loss and diabetes remission, with a lower incidence of complications compared with the conventional BPD/DS procedure (30, 31).

BPD/DS is primarily indicated for patients with a BMI ≥ 35 who present with concomitant type 2 diabetes or other components of metabolic syndrome, as well as for those who have not achieved satisfactory outcomes following other metabolic procedures including SG (32, 33). Current clinical guidelines and expert consensus generally assign BPD/DS a lower recommendation priority when compared with SG and RYGB due to its technical complexity and the necessity of balancing surgical risk against individualized weight-loss objectives.

Nutritional deficiencies and imbalances are frequently observed during follow-up in patients who have undergone BPD/DS. Deficiencies in iron, calcium, and vitamin B12 are particularly common, while deficiencies in fat-soluble vitamins including vitamins A and D, are less frequently reported (25, 34). Given the nutritional risks associated with the procedure, surgeons should initiate early communication with patients during the preoperative evaluation to fully inform them of the risks of surgical complications and the potential need for long-term postoperative nutritional monitoring and supplementation. A comprehensive preoperative risk assessment is strongly recommended prior to surgical decision-making.

### 2.4 Endoscopic bariatric and metabolic therapy

In addition to conventional surgical approaches, endoscopic bariatric and metabolic therapies (EBMTs) have emerged as minimally invasive alternatives for patients with T2DM and obesity, filling the therapeutic gap between medical management and metabolic surgery. Endoscopic sleeve gastroplasty (ESG) reduces gastric volume via endoscopic suturing, achieving moderate weight loss and significant glycemic improvement; meta-analyses report T2DM remission or improvement in approximately 50%–57% of patients at 12–24 months, with a favorable safety profile and lower perioperative risk compared with surgical procedures (35, 36). Duodenal mucosal resurfacing (DMR), another innovative endoscopic modality, targets duodenal nutrient sensing through hydrothermal ablation, improving insulin sensitivity and glycemic control independently of substantial weight loss. Clinical trials demonstrate that DMR significantly reduces HbA1c by approximately 1.0%–1.2% at 6 months, with sustained benefits up to 12 months and a low incidence of serious adverse events (37, 38). These minimally invasive procedures are particularly suitable for patients with elevated surgical risk, intermediate BMI, or reluctance to undergo surgery. As evidence accumulates, endoscopic metabolic therapies are increasingly recognized as valuable components of personalized multimodal diabetes care, warranting further investigation in large scale randomized controlled trials and long term cohort studies.

### 2.5 Factors influencing the choice of surgical procedure

In metabolic surgery for patients with diabetes, the selection of a specific surgical approach must involve a balance between safety and therapeutic efficacy. Current clinical evidence and expert consensus support the use of BMI as a primary criterion in determining the most appropriate procedure. Higher BMI levels are associated with increased surgical risk and a greater incidence of postoperative complications, including wound-related complications (39). From a patient perspective, regional variation in surgical preferences has been documented. In China, although RYGB is considered the gold standard for the treatment of T2DM and obesity based on clinical guidelines and expert consensus, only 3.1% of all metabolic surgeries performed are RYGB procedures (40). In contrast, SG, which is technically less complex, and endoscopic interventions have achieved significantly higher acceptance among patients. These trends indicate that the procedures ultimately selected are influenced not only by clinical indications but also by regional perceptions of disease and attitudes toward surgical treatment. Personalized treatment strategies remain a key determinant in the selection of surgical procedures. In addition to assessing glycemic control and preoperative metabolic status, factors including lifestyle, psychological condition, and individual understanding and perception of surgical interventions should be carefully considered.

## 3 Efficacy assessment of metabolic surgery in diabetes remission

### 3.1 Definition and criteria for diabetes remission

According to a 2009 report by the American Diabetes Association (ADA), diabetes remission is defined based on the following three core criteria: (1) discontinuation of glucose-lowering therapy (GLT); (2) maintenance of normoglycemia, typically indicated by an HbA1c level < 6.5%; and (3) persistence of this state for a minimum duration of one year (41). This definition was subsequently refined and standardized in the 2021 ADA/EASD consensus report, which provides updated criteria for remission in clinical and research settings (42). However, ongoing debate persists, as varying criteria have been used across clinical studies. These inconsistencies present challenges in the interpretation, comparison, and synthesis of clinical outcomes across investigations (43).

In clinical practice, diabetes remission is commonly classified into two categories: complete remission and partial remission. Complete remission is defined as the maintenance of HbA1c levels consistently below 6.5% for at least one year following the discontinuation of all glucose-lowering medications. Partial remission refers to either (1) achieving an HbA1c < 7% while continuing the use of GLT, or (2) maintaining HbA1c within the range of 6.5%–7% in the absence of such medications. These definitions incorporate both glycemic control, as measured by HbA1c levels, and the status of GLT as central evaluation metrics. Based on the current definitions and criteria, complete remission has been reported in up to 68% of patients within one year following metabolic surgery, with marked variation across surgical procedures (24, 44). By contrast, significantly lower remission rates have been observed with conventional medical therapy or lifestyle interventions alone (45).

### 3.2 Postoperative diabetes remission rates and influencing factors

Following metabolic surgery, the likelihood of achieving diabetes remission is influenced by several factors, including diabetes duration, preoperative glycemic control, presence of comorbidities, preoperative insulin use, and the type of surgical procedure performed. Among these, the duration of diabetes is considered a key determinant. Research has indicated that patients with a shorter duration of diabetes exhibit a significantly higher probability of achieving complete remission after surgery. In patients undergoing SG, remission rates have been significantly higher when the duration of diabetes is less than five years, compared with longer-standing disease (44). Preoperative HbA1c levels also exert a substantial influence on postoperative remission outcomes. Favorable glycemic control prior to surgery has been associated with increased rates of diabetes remission, whereas elevated preoperative HbA1c levels have been predictive of a reduced likelihood of achieving complete remission following metabolic surgery (12, 46).

In a study involving 112 patients, preoperative insulin use, along with duration of diabetes, was associated with postoperative diabetes remission outcomes (47). The presence of comorbidities including hypertension and hypercholesterolemia has also been found to negatively impact diabetes remission rates. One study demonstrated that patients with these preoperative comorbidities had significantly lower remission rates compared to those without such conditions (47, 48). By assessing these readily measurable clinical parameters, the potential efficacy of metabolic surgery for individual patients may be more accurately predicted (46).

### 3.3 Predictive indicators for diabetes remission

Several clinical scoring systems based on laboratory and clinical parameters have been shown to be useful in predicting postoperative diabetes outcomes. The ABID score, a composite index incorporating age, BMI, insulin use, and duration of diabetes has been demonstrated to effectively estimate the likelihood of diabetes remission following metabolic surgery. Lower ABID scores have been associated with higher probabilities of remission (32). The TyG index (Triglyceride-Glucose index) and HOMA-IR (Homeostatic Model Assessment of Insulin Resistance) are two widely used metabolic markers with established clinical significance. The TyG index is currently recognized as a reliable surrogate marker for insulin resistance; lower TyG index values have been associated with an increased likelihood of diabetes remission. Research has indicated that a lower baseline TyG index during preoperative assessment corresponds with a greater probability of achieving postoperative remission (49). HOMA-IR is used to quantify the degree of insulin resistance, with elevated values correlated with persistent diabetes and a reduced probability of remission following surgery. Significant postoperative reductions in HOMA-IR have been closely linked to diabetes remission. While improvements in insulin resistance are often considered a key mechanism driving remission, it remains plausible that reduced insulin resistance may instead represent a consequence of the multifactorial metabolic improvements underlying diabetes remission (50, 51).

## 4 Impact of metabolic surgery on diabetes complications and all-cause mortality

Microvascular complications induced by diabetes affect multiple organ systems and may result in conditions including diabetic nephropathy (DN), diabetic retinopathy, and diabetic neuropathy. These complications are known to significantly reduce quality of life and negatively impact long-term prognosis in patients. Metabolic surgery effectively induces diabetes remission, and sustained glycemic control achieved through such interventions significantly delays the onset and progression of diabetes-related microvascular complications. Metabolic surgery induces significant reductions in the incidence of diabetic microvascular complications. Pooled data from large cohort studies and meta-analyses demonstrate a 39% relative risk reduction in diabetic nephropathy (DN) and a 52% reduction in diabetic retinopathy following bariatric surgery. These estimates are derived from long-term observational studies with a median follow-up duration of 4.3 to 6.0 years, comparing surgical patients to matched controls receiving conventional medical therapy (lifestyle modification + pharmacotherapy) rather than baseline preoperative levels (52). In patients with pre-existing diabetic microvascular complications, metabolic surgery has been associated with reduced urinary protein excretion in those with DN and a decrease in microvascular hemorrhage in the context of diabetic retinopathy, thereby contributing to clinical improvement in both conditions (53, 54). In addition, symptoms of diabetic neuropathy have been alleviated, and regeneration of small nerve fibers has been promoted following surgery (55).

Long-term follow-up data from patients with diabetes who have undergone metabolic surgery have indicated a significantly reduced incidence of major adverse cardiovascular events in the postoperative period (56). This reduction is likely attributable to improvements in blood pressure regulation, lipid profiles, and overall metabolic homeostasis achieved following surgery. Weight loss, improved hypertension control, and normalization of lipid levels collectively contribute to a lower risk of cardiovascular events. In particular, postoperative increases in high-density lipoprotein cholesterol, along with reductions in low-density lipoprotein cholesterol and triglycerides, have been associated with attenuated atherosclerotic progression, thereby decreasing the overall cardiovascular risk (57).

A large-scale meta-analysis involving 174,772 patients demonstrated that those who underwent metabolic surgery experienced significantly improved long-term survival compared with individuals receiving conventional medical therapy. In the surgical group, a 49.2% reduction in all-cause mortality risk was observed, and median life expectancy was extended by 6.1 years when compared to the non-surgical group (58). When patients with diabetes undergoing metabolic surgery were specifically examined, the survival benefit appeared even more pronounced compared with non-diabetic patients undergoing the same procedure. In this subgroup, an approximate 60% reduction in mortality risk was reported (58).

This substantial survival advantage has been largely attributed to significant improvements in metabolic status achieved after surgery, which contribute to a reduced incidence of cardiovascular events and other life-threatening complications that adversely affect long-term prognosis in patients with diabetes (59, 60). In light of the demonstrated efficacy of metabolic surgery in controlling diabetic complications and improving long-term survival, it has been proposed that surgical eligibility criteria may be reasonably considered for expansion to include select patients with established diabetic complications. However, such expansion requires rigorous risk stratification, as certain complications-particularly those involving the cardiovascular and urinary systems-may confer substantially elevated surgical and anesthetic risks compared with patients without diabetic complications.

## 5 Long-term efficacy of metabolic surgery and the issue of diabetes recurrence

The primary measure for assessing the efficacy of metabolic surgery in patients with diabetes is the postoperative remission status of the disease. Previous retrospective studies reported that, among patients undergoing RYGB, complete remission of diabetes was achieved in approximately 57%–70% within the first year following surgery (11, 24, 47, 61). However, during long-term follow-up of up to 10 years, the rate of complete remission typically declined to around 30%, underscoring the challenge of maintaining durable glycemic control over time (62, 63). These findings indicate that diabetes remission achieved through metabolic surgery may not be permanent and can decline over time. Although patients who attain complete remission postoperatively are frequently able to discontinue antidiabetic medications initially, long-term glycemic control often necessitates the reinitiation or continued use of pharmacotherapy. Therefore, even after successful metabolic surgery, ongoing monitoring and, when indicated, the appropriate use of glucose-lowering agents remain essential to sustain metabolic improvements and prevent recurrence.

Research has demonstrated that the early postoperative administration of pharmacologic agents, including GLP-1 receptor agonists and SGLT-2 inhibitors can significantly improve diabetes remission rates and extend the duration of remission in patients who have already achieved it (64, 65). Clinical recommendations support initiation of GLP-1 RAs at 3-6 months after surgery, once gastrointestinal function has recovered and consistent oral intake is established, especially in patients with suboptimal weight loss, insufficient glycemic control, or high risk of diabetes relapse (10, 24). Dosage should be individualized and gradually titrated to minimize gastrointestinal intolerance: liraglutide may be started at 0.6–1.2 mg daily and increased to 1.8–3.0 mg daily, while semaglutide may be initiated at 0.25 mg weekly and escalated to 1.0–2.4 mg weekly as tolerated (10, 66). GLP-1 RAs act synergistically with metabolic surgery by enhancing endogenous GLP-1 signaling, suppressing glucagon secretion, reducing hepatic gluconeogenesis, and improving central glucose sensitivity, leading to more durable glycemic control and lower risk of weight regain compared with surgery alone (24, 66). For safety, GLP-1 RAs should be paused at least 7 days before any revisional surgery to reduce perioperative risk, and regular monitoring for hypoglycemia and nutritional deficiencies is advised during long term use (67). To reduce the risk of diabetes recurrence and maintain remission, comprehensive long-term postoperative management may be considered. This includes regular monitoring of glycemic and metabolic parameters, structured dietary guidance, moderate physical activity, psychological support, and continued patient education (62, 68–70). For high-risk individuals, personalized follow-up plans should be implemented, with timely access to nutritional counseling and structured exercise programs (71).

## 6 Safety and postoperative complications of metabolic surgery

Common surgical complications following metabolic procedures include infection, bleeding, and anastomotic leak—adverse events frequently encountered in abdominal surgery. In patients with diabetes, these same surgical procedures are associated with higher risks, leading to increased mortality and more severe clinical outcomes. A retrospective study involving 682 patients with diabetes who underwent metabolic surgery reported a postoperative infection rate of approximately 20%, significantly higher than that observed in non-diabetic patients (72). Another retrospective analysis identified an intraoperative bleeding incidence of up to 10% among patients with diabetes undergoing abdominal surgery, again significantly exceeding the rate documented in non-diabetic patients (67).

Anastomotic leak represents a serious and potentially life-threatening complication of abdominal surgery. Given that most metabolic procedures involve gastrointestinal reconstruction and the creation of one or more anastomoses, this risk is clinically relevant. In patients with diabetes, the presence of diabetes-related microvascular disease is known to impair tissue perfusion and healing at anastomotic sites, thereby significantly increasing the likelihood of anastomotic leaks compared with non-diabetic patients. Reported rates of anastomotic leaks in patients with diabetes undergoing RYGB have reached as high as 6.8% (73). For context, the baseline anastomotic leak rate in non-diabetic patients undergoing the same bariatric procedures ranges from 0.5% to 2.4% in large-scale meta-analyses and registry studies (72, 73).

Although postoperative complications following metabolic surgery are, to some extent, unavoidable, extensive clinical data have consistently indicated that the risk of such complications in patients with diabetes is closely associated with several preoperative factors, including age, BMI, HbA1c levels, and the presence of comorbid conditions (67, 74, 75). Identification and management of these risk factors allow for more accurate preoperative assessment of surgical risk. The tailoring of individualized operative strategies based on such assessments may be essential for effectively reducing the incidence of complications following metabolic surgery.

Since metabolic surgery primarily involves the stomach, duodenum, and jejunum, which are regions essential for the absorption of vitamins and trace elements, patients may experience long-term malabsorption of these nutrients postoperatively, potentially resulting in nutritional deficiencies (76). These effects are often persistent and, in many cases, irreversible, with the potential to significantly impair long-term prognosis. As a result, adherence to a structured nutritional management plan is required following surgery. This should include regular monitoring of serum vitamin levels, implementation of dietary modifications, and initiation of appropriate supplementation as necessary (77).

Diabetic ketoacidosis (DKA) is a serious complication that may occur in patients with diabetes following metabolic surgery, particularly among those receiving sodium-glucose cotransporter-2 (SGLT-2) inhibitors. Postoperatively, due to altered gastrointestinal anatomy and reduced oral intake during the early period of enteral nutrition, elevated blood ketone levels and disturbances in acid–base balance may develop, potentially resulting in severe clinical outcomes including coma or death (78). To reduce the risk of postoperative DKA, current clinical guidelines recommend discontinuation of SGLT-2 inhibitors prior to surgery, with reinitiation only after the resumption of a normal diet (79). Preoperative monitoring of both blood glucose and blood ketone levels is essential. In cases where elevated blood ketone levels are found, surgery should be postponed, and alternative antidiabetic therapies should be initiated.

## 7 Indications for metabolic surgery in the treatment of diabetes

In the 2016 clinical guidelines issued by the ADA, metabolic surgery was designated as a Class I indication for patients with severe obesity (BMI ≥ 35 kg/m²) and concomitant T2DM, marking the first inclusion of metabolic surgery in the treatment recommendations of the ADA. In the 2017 revision, the terminology was formally changed from “bariatric surgery” to “metabolic surgery,” reflecting a paradigm shift in the conceptualization and therapeutic objectives of the procedure. This change emphasized that the primary goal of the intervention extends beyond weight loss and is directed toward correcting metabolic dysregulation specifically to induce remission or achieve significant improvement in T2DM and other components of metabolic syndrome (80). However, despite the strength of current evidence and formal guideline endorsement, a substantial proportion of eligible patients do not undergo surgical treatment. This gap has been attributed to limited physician awareness and patient concerns regarding the surgical approach (81).

BMI remains the primary clinical criterion for determining eligibility for metabolic surgery. Traditionally, a BMI ≥ 35 kg/m² has been regarded as the standard threshold for surgical indication. However, with advancements in research, patients with lower BMI values have increasingly become the focus of clinical investigation. A retrospective study reported that, among patients with lower BMI who underwent RYGB, the rate of complete diabetes remission reached 50% within one year postoperatively (82). These findings indicate that metabolic surgery can also be effective in achieving diabetes remission in patients with lower BMI. Although this patient population tends to experience fewer postoperative complications, an increased susceptibility to nutritional deficiencies, particularly within the first postoperative year, has been observed. These include anemia and deficiencies in trace elements (83). A 2024 prospective cohort study confirmed that low-BMI patients exhibited significantly higher rates of iron deficiency (32.7%), vitamin B12 deficiency (24.1%), vitamin D insufficiency (58.3%), and calcium deficiency (18.5%) at 2-year follow-up compared to obese controls. These deficiencies were most pronounced following Roux-en-Y gastric bypass and sleeve gastrectomy, attributed to reduced absorptive surface area and intrinsic factor secretion in a smaller gastric remnant. To mitigate these risks, rigorous longitudinal monitoring is mandatory. Preoperatively, all low-BMI candidates should undergo baseline testing of serum iron, ferritin, vitamin B12, 25-hydroxyvitamin D, calcium, and parathyroid hormone. Postoperatively, quarterly monitoring for the first year and biannual assessments thereafter are recommended. Supplementation protocols include elemental iron (100–150 mg daily), vitamin B12 (1000 μg weekly), vitamin D (3000 IU daily), and calcium citrate (1200–1500 mg daily). This intensive surveillance strategy reduces clinical deficiency rates by 60% in high-risk low-BMI cohorts (84, 85). As such, thorough preoperative assessment and vigilant postoperative monitoring are essential for patients with lower BMI undergoing metabolic surgery.

Of particular relevance for Asian populations, the ADA and IDF, along with the 2022 ASMBS/IFSO global guidelines, recommend lower BMI thresholds for metabolic surgery due to earlier onset of obesity-related cardiometabolic risk. Specifically, metabolic surgery is recommended for Asian patients with type 2 diabetes at a BMI ≥27.5 kg/m² who fail to achieve adequate glycemic control with conservative management, rather than the standard BMI ≥30 kg/m² applied to other populations. This adjustment accounts for differences in body composition, fat distribution, and diabetes risk at lower BMI values in Asian individuals.

## 8 Conclusion

Metabolic surgery has been established as an effective therapeutic intervention for T2DM. It facilitates weight reduction and the correction of metabolic abnormalities, thereby improving clinical parameters, significantly decreasing the incidence of obesity-related complications, and enhancing both quality of life and long-term prognosis. Currently, the most commonly performed metabolic procedures are RYGB and SG. While these two procedures differ in surgical anatomy, they have not been definitively shown to act through distinct physiological mechanisms; instead, emerging evidence suggests they may exert shared glycemic control effects via common pathways involving modulation of the gut-brain neurological axis, particularly through vagal afferent fiber activity that regulates relevant brain centers (86). These surgical approaches vary considerably in their efficacy for managing hyperglycemia, making the individualization of surgical planning based on patient-specific characteristics essential for optimizing outcomes. Postoperative recurrence of diabetes and the development of related complications remain significant clinical challenges. Therefore, postoperative care and long-term follow-up are of critical importance. A comprehensive, multimodal management strategy should be implemented to support the maintenance of metabolic stability following surgery and to reduce the risk of disease recurrence.

Future research in the field of metabolic surgery should aim to quantitatively assess both short- and long-term outcomes and to facilitate the development of novel surgical techniques. Advancements in these areas are expected to further improve the efficacy of metabolic surgery and provide enhanced strategies and technological innovations for the prevention and treatment of chronic conditions including T2DM. Continued exploration and integration of emerging therapeutic approaches, along with ongoing refinement of existing surgical procedures and technologies, will be essential. Overall, metabolic surgery presents broad and promising potential in the management of diabetes, and ongoing research and clinical implementation are anticipated to further expand its applicability within real-world healthcare settings. Nevertheless, it is critical to acknowledge that a more ideal long term direction may not solely lie in broader surgical application, but also in fully elucidating the underlying physiological mechanisms of metabolic surgery. Such mechanistic insights could be pharmacologically replicated to achieve similar metabolic benefits with broader accessibility, lower risks, and reduced costs, representing a highly valuable complementary strategy for diabetes care in the future.
